# Identifying critical genes associated with aneurysmal subarachnoid hemorrhage by weighted gene co-expression network analysis

**DOI:** 10.18632/aging.203542

**Published:** 2021-09-20

**Authors:** Zhizhong Yan, Qi Wu, Wei Cai, Haitao Xiang, Lili Wen, An Zhang, Yaonan Peng, Xin Zhang, Handong Wang

**Affiliations:** 1 The First School of Clinical Medicine, Southern Medical University, Guangzhou 510515, China; 2 Department of Neurosurgery, Jinling Hospital, Nanjing 210002, China; 3 Department of Neurosurgery, The 904th Hospital of The Joint Logistics Support Force of Chinese People's Liberation Army, Wuxi 214000, China; 4 Department of Neurosurgery, The Affiliated Suqian First People’s Hospital of Nanjing Medical University, Suqian 223800, China; 5 Department of Neurosurgery, Kowloon Hospital, Shanghai Jiaotong University School of Medicine, Suzhou 215028, China

**Keywords:** aneurysmal subarachnoid hemorrhage, critical genes, ANXA3, ALPL, ARG1

## Abstract

Aneurysmal subarachnoid hemorrhage (aSAH) is a life-threatening medical condition with a high mortality and disability rate. aSAH has an unclear pathogenesis, and limited treatment options are available. Here, we aimed to identify critical genes involved in aSAH pathogenesis using peripheral blood gene expression data of 43 patients with aSAH due to ruptured intracranial aneurysms and 18 controls with headache, downloaded from Gene Expression Omnibus. These data were used to construct a co-expression network using weighted gene co-expression network analysis (WGCNA). The biological functions of the hub genes were explored, and critical genes were selected by combining with differentially expressed genes analysis. Fourteen modules were identified by WGCNA. Among those modules, red, blue, brown and cyan modules were closely associated with aSAH. Moreover, 364 hub genes in the significant modules were found to play important roles in aSAH. Biological function analysis suggested that protein biosynthesis-related processes and inflammatory responses-related processes were involved in the pathology of aSAH pathology. Combined with differentially expressed genes analysis and validation in 35 clinical samples, seven gene (*CD27, ANXA3, ACSL1, PGLYRP1, ALPL, ARG1,* and *TPST1*) were identified as potential biomarkers for aSAH, and three genes (*ANXA3, ALPL,* and *ARG1*) were changed with disease development, that may provide new insights into potential molecular mechanisms for aSAH.

## INTRODUCTION

Aneurysmal subarachnoid hemorrhage (aSAH) is a life-threatening medical event caused by the rupture of an intracranial aneurysm, resulting in blood leakage into the subarachnoid space [1]. According to the relevant literature, aSAH accounts for 75%-80% of nontraumatic SAH, with an annual incidence of approximately 6-16 cases per 100,000 individuals worldwide [2]. The mortality rate of SAH is estimated to be approximately 40-50%, with a 36% mortality rate within 30 days of the development of symptoms [3–5].

The most common symptom of aSAH is severe headache, usually accompanied by nausea, vomiting, photophobia, and neck rigidity [6]. Moreover, as the disease progresses, patients may also experience symptoms such as drowsiness, confusion, focal neurological deficits, hemiparesis, and coma [6]. According to previous reports, approximately 30% to 40% of the aSAH case may present with a warning headache which occurs a few weeks before hemorrhage [7–10].

The prognosis of patients with aSAH is mainly related to early brain injury, early cerebral vasospasms, and delayed cerebral ischemia, which is considered to be the main cause of unfavorable outcomes [11]. Several studies suggested that severe early brain injury after aSAH may be the leading factor contributing to death and poor prognosis in aSAH [12, 13]. Early identification of individuals with aSAH and timely adjustment of treatment are the main approaches for improving patient prognosis.

**Figure 1:**
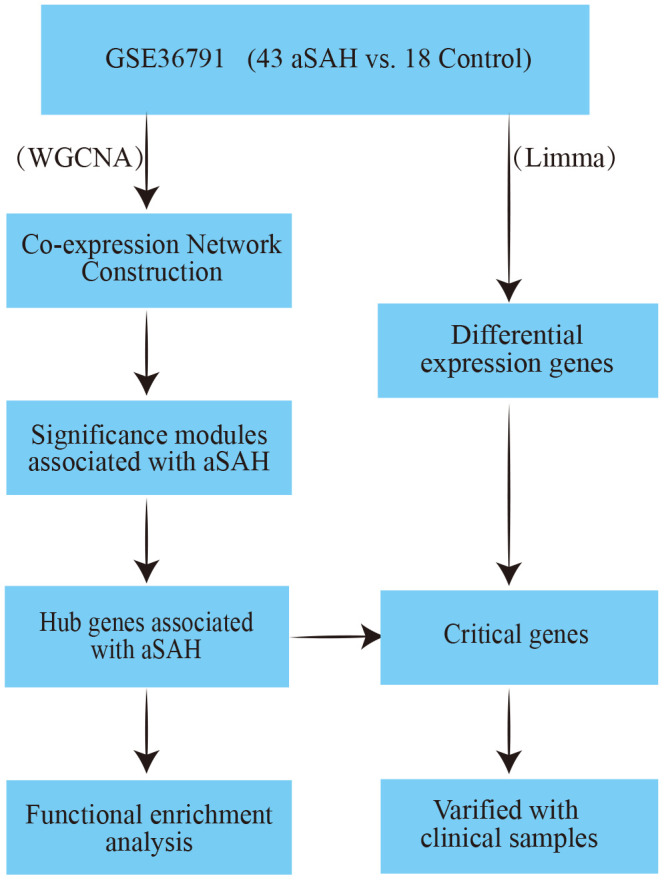
The workflow of this analysis.

In order to identify aSAH samples at an early stage, we hypothesized that potential biomarkers may exit in the peripheral blood, for prediction of aSAH. Accordingly, in this study, we analysed the peripheral blood samples of 43 patients with aSAH due to ruptured intracranial aneurysms and those of 18 individuals with headaches using the weighted gene co-expression analysis (WGCNA) to select hub genes associated with aSAH. Subsequently, critical genes were identified by combining these data with differentially expressed genes (DEG) analysis and validation in 35 clinical samples. We identified three genes, ALPL, ANAXA3 and ARG1, that may be associated with aSAH disease progression. The workflow of the current analysis is shown in Figure 1.

## RESULTS

### Data processing

For the data matrix, 12,095 unique genes were annotated with GPL10558 platform annotation file, and 7,987 of these genes were found to be protein-coding genes after referring to the human genome assembly *GRCh38.* The dataset included 43 aSAH samples and 18 control samples; n*o* outlying samples were identified with the criterion Z.k_u_ lower than the -5 base on Euclidean distance based sample network analysis. Thus, all samples expression data were applied to construct the co-expression network.

### Weighted gene co-expression network construction

**Figure 2:**
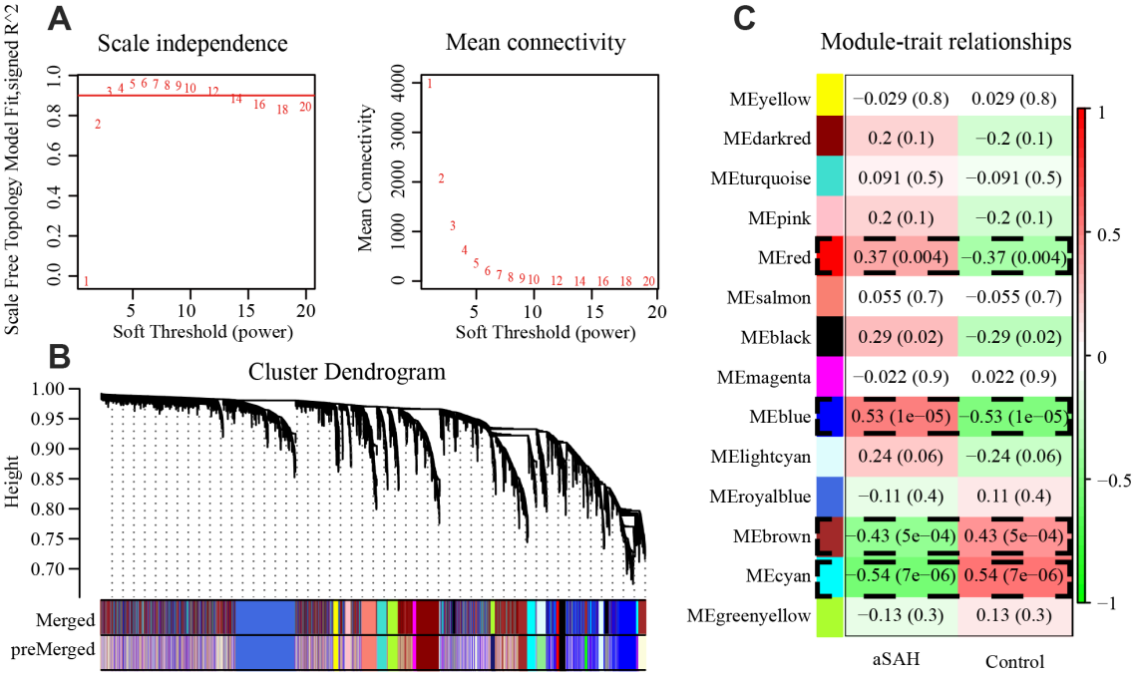
**4 modules were selected by WGCNA.** (**A**) The panel shows analysis of the scale-free fit index and mean connectivity for various soft-thresholding powers (β). (**B**) Genes with the highest median absolute deviation enriched modules in co-expression network, 20 co-expression cluster were identified after merging the high related modules with cutoff value 0.2. (**C**) Correlation between each module and phenotype. Among the modules, red (P = 0.004), blue (P = 1e-05), brown (P = 5e-04) and cyan (P = 7e-06) showed closely associated with aSAH (P < 0.01).

In WGCNA, we calculated the soft thresholding power base on the scale-free topology criterion using the pickSoft Threshold function. A beta value of 3 (R^2^ > 0.9) was chosen to construct the gene network by applying the default WGCNA approach (Figure 2A). In this analysis, 21 modules were identified with a minimum module size of 30, the medium sensitivity of 2 to branch splitting. We merged the modules with their pairwise correlation is larger than 0.8 so that to avoid modules eigengenes are highly correlated. Finally, 14 modules were picked out and they were displayed in Figure 2B.

### Identification of significant modules

**Figure 3:**
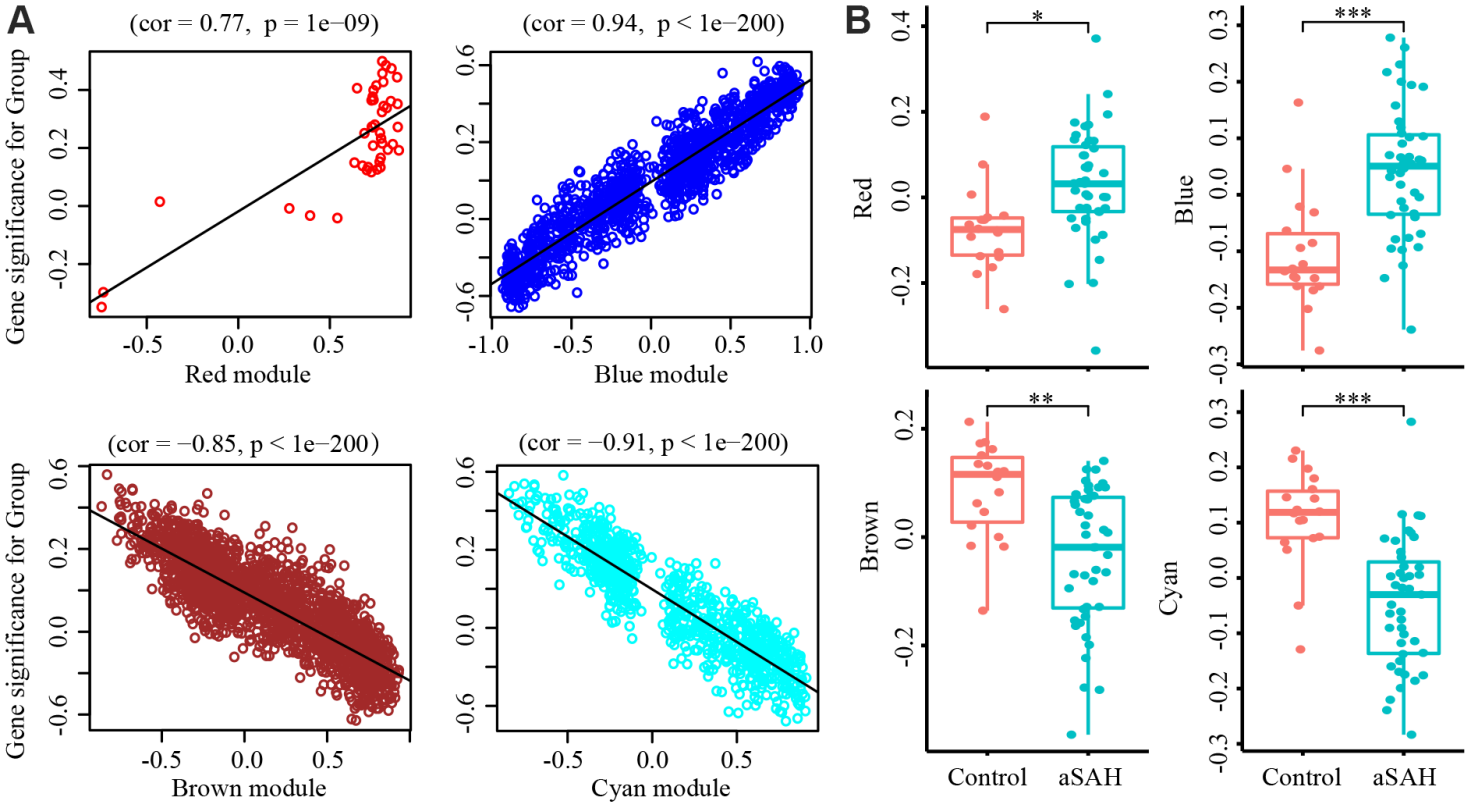
**Gene Significance (GS) and module membership (MM) were calculated with Pearson correlation analysis.** (**A**) The GS and MM are highly correlated in red (cor = 0.77, P = 1e-09), blue (cor = 0.94, P = 1e-200), brown (cor = -0.85, P = 1e-200) and cyan (cor = -0.91, P = 1e-200) module, indicated that the red and blue module contain genes that have high positive correlation with aSAH while the brown and cyan module contain genes that high negative correlations with aSAH. (**B**) The GS of aSAH group in red (*: p < 0.01) and blue (***: P < 0.0001) module were significantly higher than control group, while the opposite result was shown in the brown (**: P < 0.001) and cyan module (***: P < 0.0001).

To select modules that were significantly associated with aSAH, the association between module eigengenes and clinical characteristics was evaluated with Pearson correlation analysis. Figure 2C shows the correlations between module eigengenes and aSAH. Among the modules, red (*P* = 0.004), blue (*P* = 1e-05), brown (*P* = 5e-04) and cyan (*P* = 7e-06) were closely associated with aSAH (*P* < 0.01). Genes with a high significance for aSAH and with high module membership in the selected four modules were identified depending on the gene significance (GS) and module membership (MM) measures. GS and MM were highly correlated in red (correlation coefficient = 0.77, *P* = 1e-09), blue (correlation coefficient = 0.94, *P* = 1e-200), brown (correlation coefficient = -0.85, *P* = 1e-200), and cyan (correlation coefficient = -0.91, *P* = 1e-200) modules, indicating that the red and blue modules contained genes with high positive correlations with aSAH, whereas the brown and cyan modules contained genes with negative correlations with aSAH (Figure 3A). The gene expression of genes in the aSAH group in the red (*P* < 0.01) and blue (*P* < 0.0001) modules was significantly higher than that of the genes in the control group, whereas the opposite results was observed for the brown (*P* < 0.001) and cyan (*P* < 0.0001) modules (Figure 3B).

### Identification of hub genes and functional enrichment analysis

**Figure 4:**
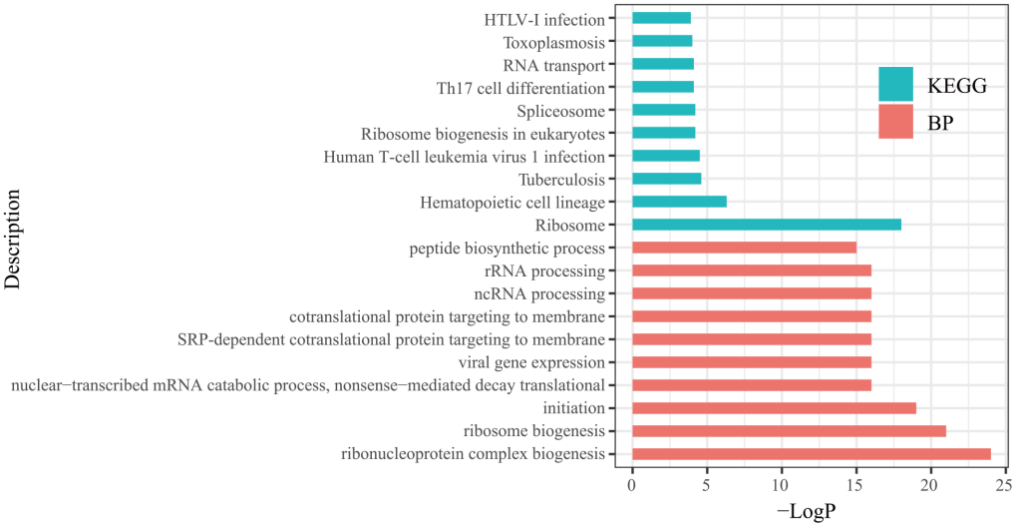
Top 10 biological processes (BP) and Kyoto Encyclopedia of Genes and Genomes (KEGG) analysis terms of hub genes.

In each significant module, hub genes were identified based on the following criteria: absolute value of the correlation between the gene and aSAH higher than 0.2 and the absolute value of the correlation of the module higher than 0.8. Based on these thresholds, 364 hub genes were identified (Table 1). Then, the hub genes list was uploaded to Metascape (http://metascape.org/) to explore the biological functions of the hub genes. The top 10 biological processes (BPs) and Kyoto Encyclopedia of Genes and Genomes (KEGG) terms annotated with hub genes are displayed in Figure 4. The BP annotations showed that the hub genes were significantly enriched in the ribonucleoprotein complex biogenesis, ribosome biogenesis, translational initiation, nuclear-transcribed mRNA catabolic process, nonsense-mediated decay, viral gene expression, SRP-dependent cotranslational protein targeting to membrane, cotranslational protein targeting to the membrane, noncoding RNA processing, rRNA processing, and peptide biosynthetic process. The KEGG pathway enrichment analysis showed that the hub genes mainly participated in ribosome, hematopoietic cell lineage, tuberculosis, human T-cell leukemia virus 1 infection, ribosome biogenesis in eukaryotes, spliceosome, Th17 cell differentiation, RNA transport, toxoplasmosis, and human T-lymphotropic virus-I infection.

**Table 1:** Hub genes in significant modules.

**Modules**	**Genes**
Red	ARRB2, CSF3R, DENND3, DYSF, GMIP, KDM6B, MAPK3, MTF1, PGD, PLEKHO2, RXRA, SEMA4A, TBC1D3G, THOC5, TOM1, USP32
Blue	ABCF1, ABHD14A, ACSL1, ADA, AIP, AKIRIN2, AKR1B1, ALPK1, ALPL, ANXA3, APEX1, ARG1, ARHGAP24, ARPC2, ATP6V0E1, ATP6V1C1, B4GALT5, BASP1, BAZ1A, BCL2, BIN1, BOLA2, BRD9, BTBD10, BUB3, C12orf57, C16orf58, CA4, CAB39, CD2, CD27, CD3D, CD7, CD81, CEBPB, CMTM6, COMMD7, CPD, CR1, CS, CSF3R, CSGALNACT2, CSNK1D, CTBS, CUTA, DCTD, DDX24, DENND3, DNMT1, DNTTIP1, DYSF, ECH1, EIF3F, EIF3G, EIF3H, EIF3K, EIF3L, ELMO2, ERP29, ESD, ESYT1, ETS2, EVI2B, EXOC6, FAM102A, FARS2, FBL, FLT3LG, FN3KRP, FPR1, FPR2, FRAT1, FYN, GK, GLO1, GNG5, GOT2, GPR141, GTDC1, GTPBP4, GZMM, HAL, HIGD2A, HINT2, HLA-DMA, HLA-DMB, IL10RA, IMP4, IMPDH2, ITGAM, ITM2A, ITPR3, ITPRIP, IVNS1ABP, JMJD8, JUNB, KCNJ15, KIF1B, KLF6, KLHL2, LAT, LAT2, LIMK2, LIN7A, LRG1, LSM2, LY9, MAL, MANSC1, MCL1, MCTP2, MEGF9, MFNG, MRPL37, MRPS24, MTMR3, NAE1, NAMPT, NDEL1, NDUFS8, NGDN, NHP2, NOL11, NOP56, NR2C2AP, NUMB, OCIAD2, OSBPL9, OSTF1, PACSIN2, PCSK7, PEBP1, PFKFB4, PGD, PGS1, PHF21A, PHTF1, PLSCR3, PLXNC1, POLR1E, POLR2H, PPIH, PRKCD, PRMT1, PTEN, PVRIG, PYGL, QPCT, RANBP1, RASGRP4, RCC2, REPS2, RFTN1, RNF149, RNF24, RNPS1, ROPN1L, RPL10A, RPL12, RPL15, RPL18A, RPL19, RPL22, RPL24, RPL3, RPL35, RPL36, RPL5, RPL6, RPS16, RPS20, RPS27, RPS27A, RPS29, RPS3, RPS4X, RUVBL1, SAMSN1, SCAMP3, SF3A3, SH3GLB1, SLA, SLC25A44, SLC9A8, SMAP2, SNRPB, SNRPF, SP100, SPAG9, SPI1, SRGN, SSBP4, STAT3, STX3, STXBP5, TGFA, TLR4, TLR8, TMED3, TMEM109, TMEM160, TMEM203, TNFRSF1A, TNFRSF25, TPST1, TRIB1, TRIM25, TUBB, UFC1, URM1, USP32, VNN3, XRCC6, ZFAND3, ZNF281, ZNF428
Brown	ABHD14A, ACAD9, ACTR5, AFG3L2, ALKBH3, BRD9, C16orf58, C8orf33, CCNDBP1, CD320, COPS6, CSE1L, CTNNBIP1, CTNNBL1, DCTD, DDX54, DNAJC9, DUS1L, EBP, ECHS1, EIF2B4, EIF3L, ELAC2, ENO2, ERP29, EXOSC1, EXOSC5, FARS2, FLT3LG, FNBP4, FXYD5, GGA2, GNL2, GRWD1, GSS, HIC2, IDUA, ITFG2, LCMT1, LONP1, LTBP3, LZTR1, MDC1, MFSD3, MORC2, MPRIP, MPV17, MRPL11, MRPL12, MRPL2, MRPL38, MRPS9, N6AMT1, NFX1, NHP2, NOP2, NUBP1, NUDC, NXT1, PAAF1, PACS2, PDCL3, PDXP, PEX14, PHB, PIGP, PLSCR3, POMT1, PRMT7, PRPF31, PTRH1, RASSF1, RNF220, RNF26, RPAIN, RPL19, RSAD1, SDHAF1, SIGMAR1, SLC25A1, SLC7A6, SNRPB, TACO1, TEX10, TMEM109, TMEM147, TMEM41A, TNFRSF25, TOMM40, TRIM68, TRMT1, TTC31, TYSND1, UCKL1, VAC14, WDR18, WDR74, ZBTB9, ZC3HC1, ZDHHC14, ZFP90, FYVE16
Cyan	ACTR1A, AKAP11, API5, BRIX1, BUB3, C11orf1, C16orf58, CA4, CCND2, CD2, DDX47, DNAJC9, DNMT1, DOCK10, ETS1, FNBP4, GIMAP6, GOLGA8B, GTPBP4, HADH, HIBADH, IL7R, ITK, KHDRBS1, KIFAP3, LANCL1, LRIG1, LY9, MATR3, MPHOSPH10, NAE1, NOL11, NSA2, NUP54, PDCL3, PDLIM7, PEBP1, PGLYRP1, PPP3CC, PSMG2, PVRIG, RALGAPA1, RFTN1, RPL15, RPL22, RRN3, SET, SH2D1A, SLC7A6, SMARCAD1, STAMBPL1, SUCLG2, TARBP1, TC2N, TFB2M, THOC1, THUMPD1, TRAT1, TUBB, UBE2N, UBE2Q2, WBP11, WWP1, XPO4, ZNF529

### Identification and validation of critical genes

**Figure 5:**
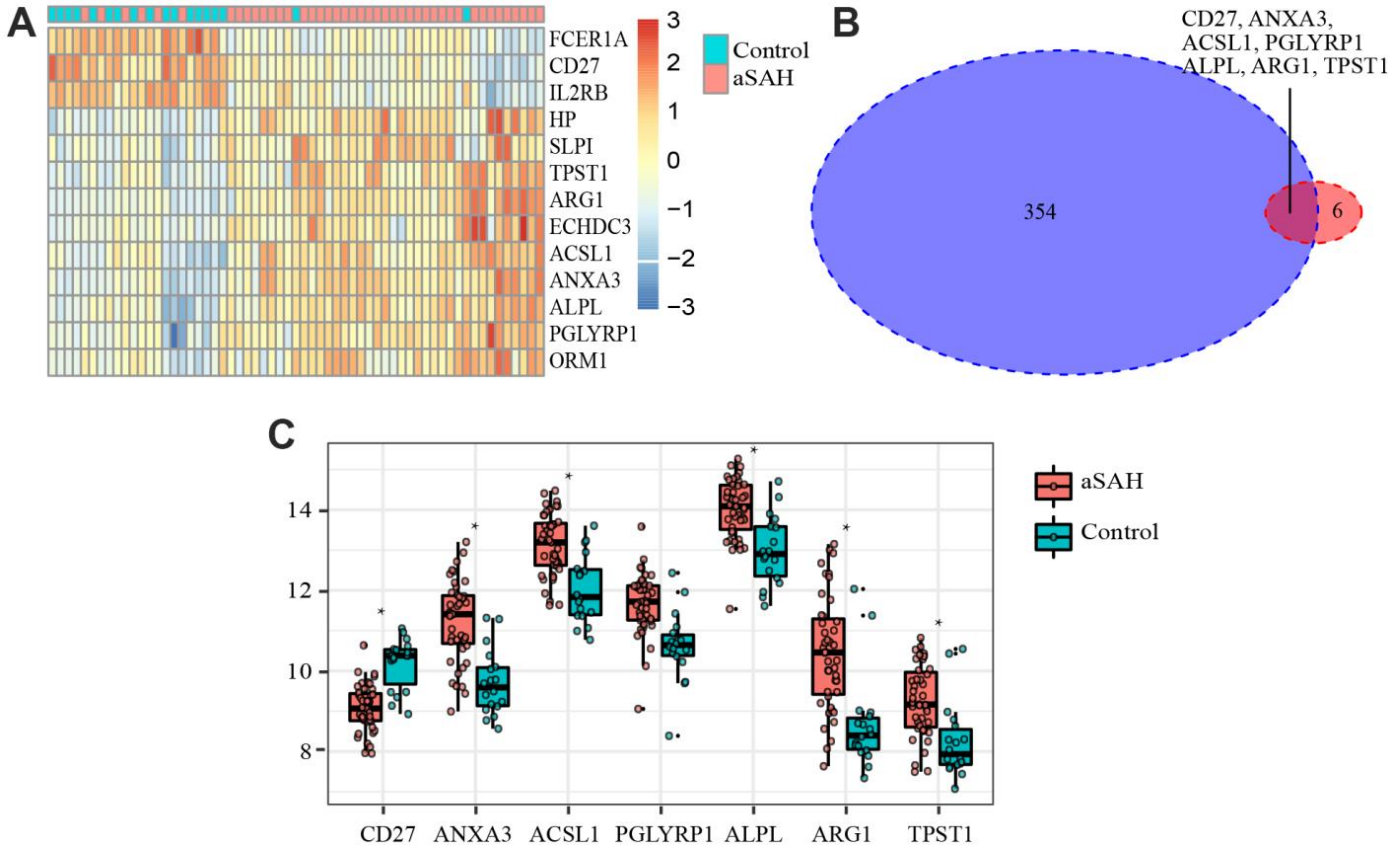
**Differentially expressed genes (DEGs) and critical genes.** (**A**) Heatmap of DEGs. (**B**) 7 critical genes were selected. (**C**) Expression of 7 critical genes in GSE36791.

**Figure 6:**
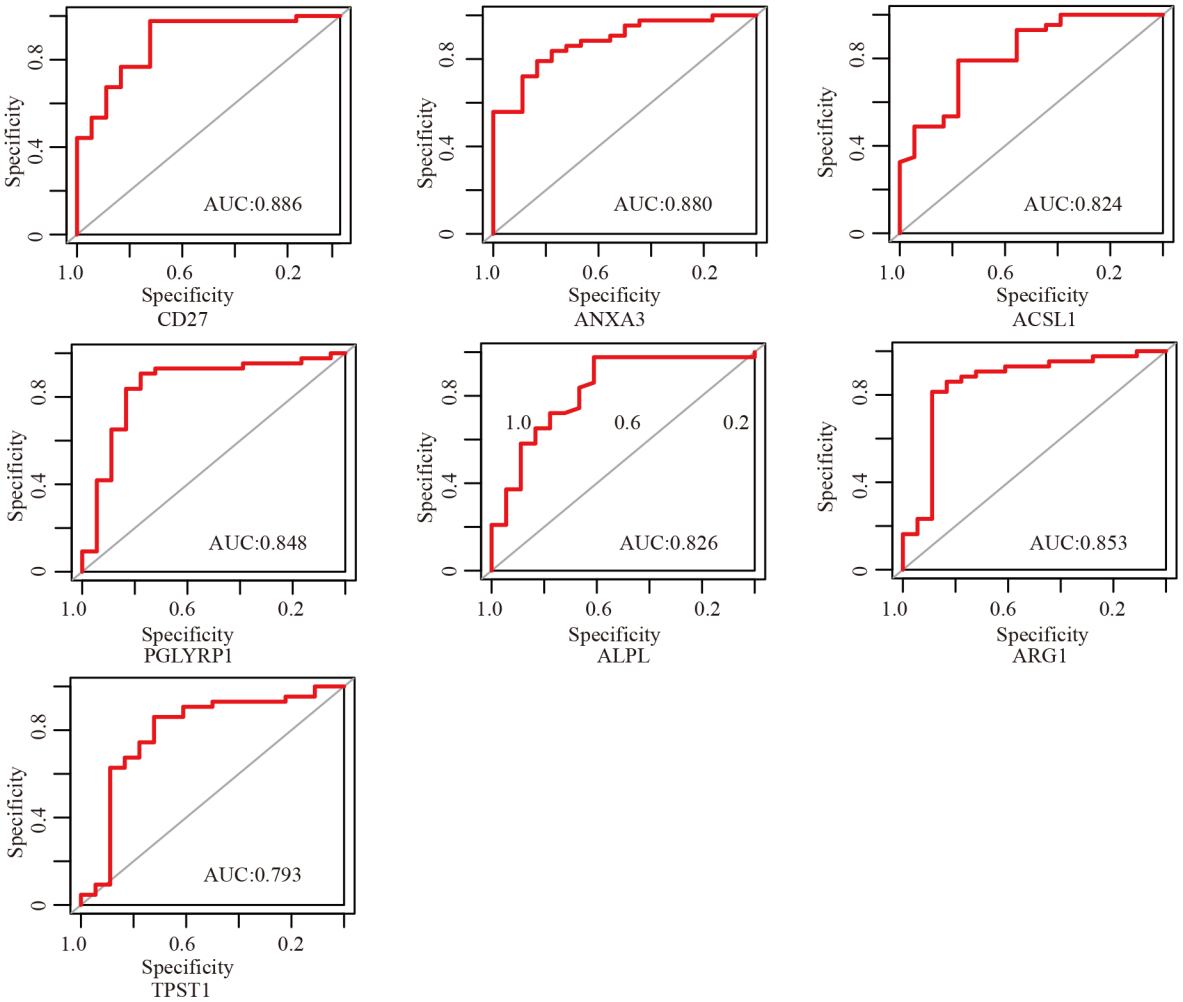
**ROC curves of critical genes.** The AUC of almost all critical gene was higher than 0.8.

In order to select critical genes from the hub genes, we analyzed the DEGs between aSAH and control individuals using the limma package, according to the cut-off criteria of |log_2_ fold change (FC)| greater than or equal to 1 and adjusted *P* value less than 0.05. Among all genes, 13 genes (*CD27, IL2RB, FCER1A, ANXA3, ACSL1, HP, PGLYRP1, ALPL, ARG1, TPST1, SLPI, ECHDC3*, and *ORM1*) were screened as DEGs (Table 2). The expression profile heatmap of the DEGs is shown in Figure 5A. Among the genes, 7 overlapped genes (*CD27, ANXA3, ACSL1, PGLYRP1, ALPL, ARG1,* and *TPST1*) were identified between DEGs and hub genes, including in one down-regulated gene (*CD27*) and six up-regulated genes (*ANXA3, ACSL1, PGLYRP1, ALPL, ARG1*, and *TPST1*) in aSAH (Figure 5B, 5C). These genes were defined as critical genes with playing a key role in the aSAH development. Receiver operating characteristic (ROC) curve was plotted and the area under the curve (AUC) was calculated to distinguish individuals with aSAH from controls. The AUCs of almost all critical gene was higher than 0.8 in the datasets, indicated they may be act as potential biomarker in diagnosing aSAH (Figure 6).

**Table 2:** DEGs identified with limma package.

	**LogFC**	**AveExpr**	**t**	**P.Value**	**Adj.P.Val**	**B**	**Change**
CD27	-1.10594	9.404925	-6.84003	3.87E-09	7.72E-06	10.67715	Down
IL2RB	-1.0878	9.875714	-6.45442	1.80E-08	2.37E-05	9.234499	Down
FCER1A	-1.49737	9.451985	-6.38427	2.37E-08	2.37E-05	8.973335	Down
ANXA3	1.525969	10.78493	5.864546	1.82E-07	5.68E-05	7.056242	Up
ACSL1	1.08668	12.80813	5.031488	4.35E-06	0.000395	4.081304	Up
HP	1.29243	9.336274	4.982566	5.22E-06	0.000414	3.911711	Up
PGLYRP1	1.053254	11.36885	4.885738	7.46E-06	0.000505	3.578054	Up
ALPL	1.032425	13.71477	4.861184	8.16E-06	0.000537	3.49388	Up
ARG1	1.751164	9.920118	4.756838	1.19E-05	0.000649	3.138231	Up
TPST1	1.001745	8.967003	4.088617	0.000126	0.003169	0.950828	Up
SLPI	1.003622	9.238332	4.083136	0.000128	0.003208	0.933614	Up
ECHDC3	1.008504	8.890846	3.535259	0.000772	0.010721	-0.7168	Up
ORM1	1.069796	10.33428	2.994054	0.003936	0.033948	-2.19028	Up

**Figure 7:**
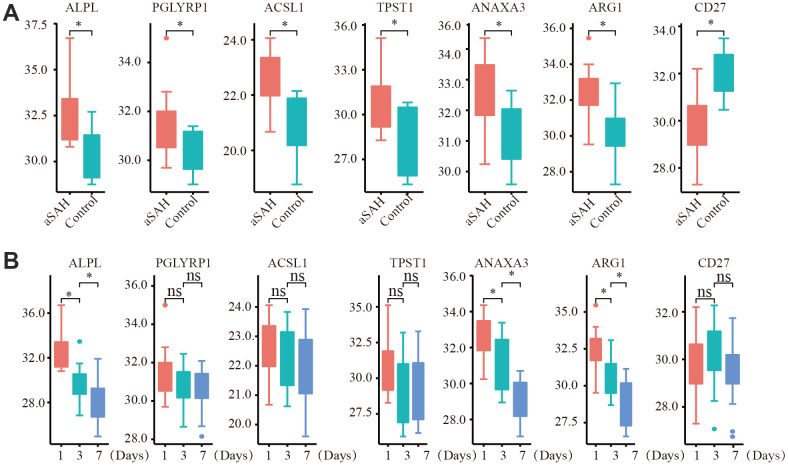
**The expression of critical genes tested by qRT-PCR with clinical data.** (**A**) ANXA3, ACSL1, PGLYRP1, ALPL, ARG1, TPST1 were obviously up regulated in aSAH patients, while CD27 was down regulated. (**B**) The expression of ALPL, ANAXA3, and ARG1 were obviously reduced over time. (*: p < 0.05).

To further test our analysis, we detected the expression of critical genes based on clinical data. The expression of the selected critical genes was significantly different between the aSAH and control groups (Figure 7A). *ANXA3, ACSL1, PGLYRP1, ALPL, ARG1,* and *TPST1* were obviously up regulated in patients with aSAH, whereas *CD27* was down regulated, the results were consistent with our analysis. We also detected the gene expression in patients with aSAH at 3 days and 7 days after diagnosis. Interestingly, the expression levels of *ALPL, ANAXA3,* and *ARG1* were reduced over time (Figure 7B).

## DISCUSSION

aSAH accounts for 75%-80% of all cases of SAH and is associated with a high mortality rate of approximately 40–50%, with a 30-day fatality rate of 36% [2, 4, 5]. The molecular mechanisms involved in the pathophysiology of aSAH remain unclear. Therefore, exploring susceptibility modules and genes for aSAH may contribute to the early diagnosis and treatment of SAH, thereby reducing mortality and serious adverse reactions.

In this study, four modules were found to be highly associated with aSAH, based on WGCNA. Additionally, 364 hub genes were identified. BP and KEGG enrichment analyses suggested that protein biosynthesis-related processes and inflammatory response-related processes were significantly involved in the pathology of aSAH. Among the hub genes, seven were found to be differentially expressed between aSAH and control groups and were identified as critical genes involved in the development of aSAH, with potential applications in the early prediction of aSAH. Finally, we validated our results using clinical data obtained from quantitative reverse transcription polymerase chain reaction (qRT-PCR).

Among the critical genes identified in this study, *CD27, ANXA3, PGLYRP1,* and *ARG1* are closely associated with the immune system. CD27, a transmembrane glycoprotein, plays an important role in immune response and is expressed on most B lymphocytes cells, T lymphocytes cells, and natural killer (NK) cells [14, 15]. The expression of CD70, which is primarily controlled by antigen receptor and Toll-like receptor stimulation on T cells, B cells and dendritic cells is a key factor in determining the contribution of CD27 to the immune response [16]. CD27 binds to the receptor CD70, and plays important roles in regulation of the activation of T lymphocytes cell and the synthesis of immunoglobulin. In this study, CD27 was obviously down regulated in the aSAH group with that in the control group, suggesting that decreased *CD27* transcription was related to B lymphocytes, T lymphocytes, and NK cells in patients with aSAH. This finding was consistent with the result from Joanna et al., who revealed that decreased *CD27* mRNA expression was related to T lymphocytes in aSAH [17]. *ANXA3, PGLYRP1,* and *ARG1* were all participated in the recognition of bacteria by neutrophils. *ANXA3* is particularly abundant in neutrophils, accounting for approximately 1% of all cytosolic proteins [18] and contributing to neutrophil antimicrobial activity by promoting phagolysosome fusion [19]. PGLYRP1, which belongs to a family of PGN-binding proteins (PGRPs), highly conserved among insects and mammals, is an antibacterial protein found in neutrophil tertiary granules. *PGRP1* plays critical roles in neutrophil production of reactive oxygen species and modulation of immune response [20, 21]. *ARG1* is stored in granules of neutrophils. Once released and activated, *ARG1* can degrade extra cellular arginine, resulting in inhibition of the activation and proliferation of T lymphocytes cells [22]. We found that *ARG1* was significantly upregulated in the SAH group, suggesting that high expression of *ARG1* may be related to decreased T lymphocytes activation and proliferation in patients with aSAH. Therefore, these results suggested that there was an abundance of transcripts related to monocytes and neutrophils with a simultaneous decrease in transcripts related to T lymphocytes in patients with aSAH.

*ACSL1* is an enzyme that converts free long-chain fatty acids into fatty acyl-CoA esters, and thereby plays critical roles in both in lipid biosynthesis and fatty acid degradation [23]. Thus, disordered lipid metabolism may be involved in the development of aSAH. A previous study also demonstrated that related membrane lipid metabolism is altered in spastic basilar arteries after SAH [24]. Statins have been used and show significant benefits in models of traumatic brain injury and the related disease processes, including cerebral ischemia, intracerebral *haemorrhage,* and *SAH* [25]. The most compelling preclinical data has been obtained in experimental SAH, where statins have been shown to reduce vasospasm and improve outcomes after SAH in the animal experiments [26–28]. Similarly, statin treatment has been shown to improve outcomes in murine models of intracranial hemorrhage [29, 30] and acute ischemic stroke [31–33]. *ALPL* encodes tissue-nonspecific alkaline phosphatase (ALP), which has key roles in skeletal mineralization via the regulation of diphosphate levels. *ALP* can also promote vascular calcification by catalyzing the hydrolysis of organic pyrophosphate, an inhibitor of vascular calcification [34]. A number of studies have reported that a close relationship between serum *ALP* and increased morbidity and mortality in patients with cardiovascular diseases [35, 36]. Moreover, elevated serum *ALP* levels have been shown to be associated with increased mortality rates, poor functional outcomes, and disease recurrence in patients with stroke [37, 38]. Zhu et al. evaluated the association between the outcomes and serum *ALPL* level in 196 patients with aSAH and found that higher serum *ALP* levels are associated with an increased risk of vasospasm, delayed cerebral ischemia-induced clinical deterioration, and functional outcomes after aSAH [39]. Thus, ALPL may be a predictive biomarker for patients with aSAH.

*TPST1*, a type of homologous tyrosyl protein sulfotransferase (TPST) enzymes, plays a critical role in protein tyrosine sulfation for transfer of sulfate from the cofactor PAPS (3'-phosphoadenosine 5'-phosphosulfate) to a context-dependent tyrosine in a protein substrate [40]. To date, the functional importance of protein tyrosine sulfation is still unclear; however, this process has been shown to play a role in altering biological activities of proteins, modulating the proteolytic processing of bioactive peptides [41], influencing the half-life of proteins in circulation [42], and regulating extracellular protein-protein interactions, as observed for inflammatory leukocyte adhesion [43, 44]. The recent discovery of tyrosine sulfation of chemokine receptors suggests an even broader role in inflammatory responses [45, 46]. The role of *TPST1* in aSAH is unknown, and further studies are needed to explore the potential mechanisms.

With the development of sequencing technology, genomics is playing an important role in disease diagnosis, mechanism research, drug development and treatment, especially in tumor diagnosis and treatment. Nowadays, sequencing technologies are increasingly used in clinical settings, and key genes may play an important role in the occurrence and development of a certain disease. Therefore, the expression of key genes can be used to determine the diagnosis of aneurysm, and the intervention of key genes can be used to treat aneurysm, thus preventing the occurrence of serious complications.

In conclusion, in this study, we found that protein biosynthesis-related processes and inflammatory responses related processes were involved in the pathology of aSAH. Additionally, we found that *CD27, ANXA3, ACSL1, PGLYRP1, ALPL, ARG1,* and *TPST1* were significant potential biomarkers to guide the identification and treatment of aSAH. According to our PCR data, the levels of *ALPL, ANAXA3*, and *ARG1* were reduced over time in patients with aSAH. However, further studies are needed to determine the relationships of these changes with the disease status. Moreover, our study lacked extensive clinical experimental verification of the identified genes. Thus, in future analyses, it will be necessary to verify our findings in clinical studies.

## MATERIALS AND METHODS

### Microarray data processing

The GSE36791 gene expression matrix was retrieved and obtained from the Gene Expression Omnibus (GEO) (https://www.ncbi.nlm.nih.gov/geo/) by using the GEO query package in the R environment (Version 4.2.0) [47]. Gene expression data from peripheral blood samples were obtained from 43 patients with SAH caused by a ruptured intracranial aneurysm and 18 patients with headaches symptoms as the control group, the detail characteristics of all samples were displayed in Table 1 of the paper-Gene expression profiling of blood in ruptured intracranial aneurysms: in search of biomarkers [17]. The corresponding annotation file-GPL10558 matrix which includes more than 47,000 probes and targets to more than 31,000 annotated genes, was downloaded and applied to convert the probe into the target gene. If the target gene was annotated with two or more probes, the mean value was calculated for subsequent analysis. Among the targeted genes, the protein-coding genes were obtained by referring to the human genome assembly *GRCh38*. The matrix was normalized without transformation by using the Bead Array package [48]. In this analysis, the data were log2 transformed. The outlying microarray samples were identified with Euclidean distance-based sample network methods and a Z.k_u_ cut-off of -5 was calculated as ku-mean(k) / sqrt(var[k]) [49].

### Weighted gene co-expression network construction

WGCNA was performed to identify clusters that were highly correlated with all three phenotypes using the WGCNA package [49]. First, the soft threshold beta was chosen via scale free topology with the R function pickSoft Threshold. Second, we applied a power adjacent function to select adjacencies between all protein-coding genes and to transform data into a topological overlap matrix (TOM), and the corresponding dissimilarity (1-TOM) was evaluated. Third, the parameters of cutree Dynamic function were set as a minimum gene size of 30 and a medium sensitivity of 2 for branch splitting to calculate the average linkage hierarchical clustering tree. Finally, highly correlated modules were merged with a pairwise correlation coefficient higher than 0.8 for the identification of modules with very similar expression profiles depending on the clustering methods.

### Identification of significant modules

To select modules that were significantly associated with aSAH, the associations between module eigengenes and aSAH were evaluated via Pearson correlation analysis. The modules with the *P*-value < 0.01 were considered to be significantly associated with aSAH. Since the module eigengene is an optimal summary of the gene expression profiles of a given module, it is natural to correlate eigengenes with these characteristics and to find the most important associations. To quantify the similarity of all genes on the array to the identified module. We quantify associations of individual genes with aSAH by defining GS as the absolute value of the correlation between the gene and the specific trait and by defining the quantitative measure of MM as the correlation of the module eigengene and the gene expression profile.

### Identification of hub genes and functional enrichment analysis

In each significant module, hub genes were screened according to the following criterion, including the absolute value of the correlation between the aSAH and gene higher than 0.2 and the absolute value of the correlation of the module higher than 0.8 [50]. To explore the biological function of the hub genes, we performed Gene Ontology (GO) and KEGG enrichment analysis using Metascape (http://metascape.org/) [51]. The top 10 GO terms and KEGG terms were visualized with the ggplot2 package in the R programming language [52].

### Identification of critical genes

In this analysis, the critical genes were identified based on two traits: significant differential expression between aSAH and control samples and high interconnections with genes in the module. Briefly, critical genes were defined as differentially expressed hub genes. The limma package [53] was applied to identify differential expressed genes (DEGs) between two groups in the expression data with the cut-off criteria |log_2_ fold change (FC)| ≥ 1 and adjust *P* value < 0.05 [54]. Then, the critical genes were screened and visualized with the Venn diagrams package [55]. The expression of critical genes was displayed and they were verified in another dataset. Additionally, ROC curves were plotted with the pROC package to verify the diagnostic performance of critical genes.

### Validation of critical genes using qRT-PCR

Finally, we validated the obtained results from microarray data of peripheral blood samples by using qRT-PCR on samples from 25 patients with aSAH and 10 healthy controls recruited from the Department of Neurosurgery, Jinling Hospital, the First School of Clinical Medicine, Southern Medical University, China. Blood was collected from patients with aSAH at three time points: before therapy, 3 days after aSAH, and 7 days after aSAH, and that from the control samples was collected at the physical examination center. All samples were obtained in the fasting condition. The characteristics of the recruited patients are shown in Table 3. Whole blood samples were homogenized in TRIzol reagent (Servicebio, Wuhan, China). Total cellular RNA was then extracted and transcribed into cDNA using a Servicebio RT First-strand cDNA Synthesis Kit (Servicebio, Wuhan, China). qPCR was subsequently performed by using 2×SYBR Green qPCR Master Mix (Low ROX; Servicebio, Wuhan, China) with the CFX Real-time PCR system (Bio-Rad Laboratories, MN, USA). Table 4 lists all primer oligos, which were synthesized by Servicebio Biotechnology (Wuhan, China). The mRNA levels of glyceraldeyhyde 3-phosphate dehydrogenase (GAPDH) were used for normalization of mRNA expression (The average qRT-PCR values are shown in Supplementary Table 1). Subsequently, relative quantification was performed based on the comparative threshold cycle (2^-ΔΔCT^) method. The qPCR experiment of each clinical sample was repeated for 3 times, and the mean value were calculated for differential comparation. The differential gene expression between the two groups was analyzed using a non-parametric test, and *P* values less than 0.05 were considered statistically significant.

**Table 3:** The characteristics of recruited patients.

**Characteristics**	**aSAH**	**Control**
Age	64.62 ± 12.26	63.25 ± 13.54
Male/Female	14/11	6/4
Hypertension	72.00 %	70.00%
Diabetes	32.00%	30.00%
Stroke (cerebral ischemia)	30.00%	20.00%

**Table 4:** Primer sequences of critical genes used in this study.

**Critical genes**	**Direction**	**Primer sequences**
ARG	Forward primer	TGGCAAGGTGATGGAAGAAAC
	Reverse primer	TCCCGAGCAAGTCCGAAAC
CD27	Forward primer	CTGTCGGCACTGTAACTCTGGTC
	Reverse primer	TCAGCGAAGGGTTTGGAAGAG
ANXA3	Forward primer	GCTGAAAGATGACTTGAAGGGTG
	Reverse primer	CCTTCATTTGCCTGCTTGTCC
ACSL1	H-ACSL1-S	CCCATGAGCTGTTCCGGTATT
	H-ACSL1-A	ACCCGCCACTTCCACTGACT
PGLYRP1	Forward primer	GAGCCTGCCCTTACGCTATGT
	Reverse primer	ACGAGCCCGTCTTCTCCAAT
ALPL	Forward primer	AAGGACGCTGGGAAATCTGTG
	Reverse primer	CGTCAATGTCCCTGATGTTATGC
TPST1	Forward primer	CCAAGTAATCAAGCCAGTCAATG
	Reverse primer	GTTGGAATTCTCCCTTATAGACCCT

### Data availability

The data used to support the findings of this study are from previously reported studies and datasets, which have been cited.

## Supplementary Material

Supplementary Table 1
