# Supplementary material for: Identifying critical genes associated with aneurysmal subarachnoid hemorrhage by weighted gene co-expression network analysis
Source: Aging (Albany NY). 2021 Sep 20;13(18):22345–60. doi: 10.18632/aging.203542 (PMC8507255; doi:10.18632/aging.203542)
Supplement: Supplementary Table 1 [file aging-13-203542-s001.pdf]

SUPPLEMENTARY TABLE

Supplementary Table 1. The average qRT-PCR values.

Genes	Control	aSAH (1 day)	3 days	7 days
ALPL	30.55±1.46	32.42±1.49	29.77±1.42	27.96±2.02
PGLYRP1	30.35±0.89	31.57±1.37	30.91±0.98	30.64±1.04
ACSL1	21.01±1.30	22.76±0.90	22.27±1.01	21.92±1.28
CD27	32.03±0.97	29.86±1.37	30.20±1.33	29.58±1.31
TPST1	28.12±2.30	30.83±1.70	29.23±2.26	29.46±2.17
ANXA3	31.08±1.09	32.52±1.23	31.17±1.47	29.08±1.08
ARG1	30.14±1.81	32.40±1.27	30.59±1.39	28.80±1.56
